# Amino Acid-Linked Low Molecular Weight Polyethylenimine for Improved Gene Delivery and Biocompatibility

**DOI:** 10.3390/molecules25040975

**Published:** 2020-02-21

**Authors:** Xiao-Ru Wu, Ji Zhang, Ju-Hui Zhang, Ya-Ping Xiao, Xi He, Yan-Hong Liu, Xiao-Qi Yu

**Affiliations:** Key Laboratory of Green Chemistry and Technology (Ministry of Education), College of Chemistry, Sichuan University, Chengdu 610064, China; kaopubro@163.com (X.-R.W.); jhzhang429@163.com (J.-H.Z.); 15982103680@163.com (Y.-P.X.); seacyhe@163.com (X.H.); yanhongliu@scu.edu.cn (Y.-H.L.)

**Keywords:** non-viral gene vector, polycations, gene delivery, biodegradability

## Abstract

The construction of efficient and low toxic non-viral gene delivery vectors is of great significance for gene therapy. Herein, two novel polycations were constructed via Michael addition from low molecular weight polyethylenimine (PEI) 600 Da and amino acid-containing linkages. Lysine and histidine were introduced for the purpose of improved DNA binding and pH buffering capacity, respectively. The ester bonds afforded the polymer biodegradability, which was confirmed by the gel permeation chromatography (GPC) measurement. The polymers could well condense DNA into nanoparticles and protect DNA from degradation by nuclease. Compared with PEI 25 kDa, these polymers showed higher transfection efficiency, lower toxicity, and better serum tolerance. Study of this mechanism revealed that the polyplexes enter the cells mainly through caveolae-mediated endocytosis pathway; this, together with their biodegradability, facilitates the internalization of polyplexes and the release of DNA. The results reveal that the amino acid-linked low molecular weight PEI polymers could serve as promising candidates for non-viral gene delivery.

## 1. Introduction

Gene therapy is a promising therapeutic method for delivering nucleic acid agents or genome-editing tools into diseased cells [1]. Initial research focused on the development of viral vectors including adenoviruses and retroviruses which have high efficiency toward delivering nucleic acids [2]. However, what troubles us is the toxicity, immunogenicity and limitations of scaling up these procedures [3]. Thus, one of the great challenges of gene delivery is to design novel non-viral vectors that fulfill the promise of high delivery efficiency and high safety [4]. Various non-viral systems, such as cationic liposomes [5], polymers [6], inorganic nanoparticles [7] and quantum dots [8], have been developed to improve the gene delivery properties. Among these types, cationic polymers have attracted great attention because of some remarkable advantages including facile manufacturing, high nucleic acid capacity, good stability and easily modification [7].

Polyethylenimine (PEI) is the most studied cationic polymer for gene delivery, and branched PEI 25 kDa has been considered as the golden standard for new polymeric non-viral vectors on account of its high transfection efficiency (TE), which is attributed to the strong buffering capacity in the pH range of 7.4–5.1 [9]. Although high molecular weight PEI shows good TE, it also exhibits obvious cytotoxicity for its non-degradable and highly positively charged structure [10,11]. Reducing molecular weight could solve the toxicity problem, but the TE is sacrificed [12]. To reduce surface charge and prolong the circulation time of cationic nanoparticles, polyethylene glycol (PEG) or other hydrophilic chains have been introduced to high molecular weight PEI [13]. On the other hand, low molecular weight (LMW) PEI could also be connected by degradable linkers including ester [14], disulfide [15] and β-aminoester [16] to achieve enhanced TE and reduced toxicity. Beside PEI, another kind of classical polycation is poly-_L_-lysine (PLL) [17], which can get rid of biological toxicity and system side effects for its polypeptide chain connected by amide bond that can be degraded by an internal enzyme [18]. However, since all amine groups in PLL are protonated primary amines [19], PLL has no pH buffering capacity at physiological environment. Meanwhile, histidine can be cited as a modification group in non-viral gene vectors for its good pH buffering capacity from the imidazole ring, especially in the more acidic endosomes and lysosomes [20].

Recently, we developed some polycations via Michael addition between LMW PEI 600 Da and diacryl esters, and these materials exhibited promise as non-viral gene vectors with higher TE and lower toxicity compared to PEI 25 kDa [21]. Herein, we combine the advantages of cationic PEI and amino acids with their special functions. The designed polycations were constructed from PEI 600 Da and linkages modified by amino acids such as lysine and histidine. Lysine can strengthen the DNA binding ability of the vector, while histidine has the potential to improve the pH buffering capacity. The low molecular weight of PEI and the proteinogenic common amino acids gave the polymers much lower cytotoxicity. Results demonstrated that the title vectors were able to condense DNA into nanoparticles with good stability. Furthermore, these materials showed higher TE and lower toxicity comparing to PEI 25 kDa whether with the presence of serum or not. The results reveal that such cationic polymers could serve as promising candidates for non-viral gene delivery.

## 2. Results and Discussion

### 2.1. Synthesis and Characterization of Target Polymers

**Scheme 1:**
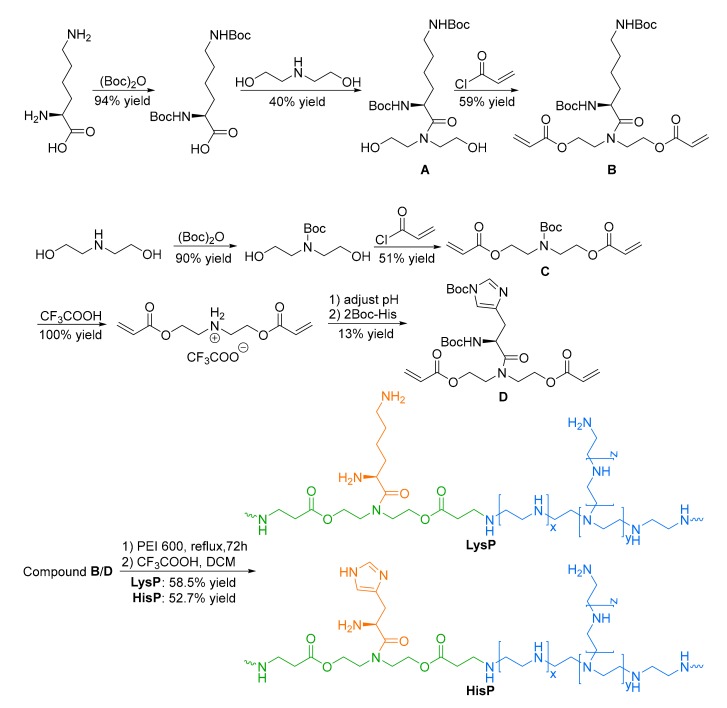
Synthetic route of cationic polymers.

We herein adopted a strategy of crosslinking PEI 600 Da via Michael addition polymerization to form degradable polymers containing nontoxic amino acid-containing bridges. For their special basicity and pH buffering capacity of lysine and histidine respectively, these two amino acids were introduced to the bridge to crosslink LMW PEI. The preparation routes of target polymers **LysP** and **HisP** are shown in Scheme 1. The Boc-protected amino acids were coupled with diethanolamine via amide condensation to give relevant bridges **B** and **D**. It is worth mentioning that different reaction sequences were applied. For bridge **B**, di-Boc-lysine could react with diethanolamine to give the coupling product **A**, which subsequently reacted with acryloyl chloride. Meanwhile, in the preparation of **D**, diethanolamine must be acrylated first, otherwise the coupling would not smoothly happen. Then, Michael addition polymerization took place between the bridges **B**/**D** and PEI 600 Da with the mole ratio of 1:1 in anhydrous dichloromethane and methanol at 45 °C for 72 h. After the reaction, trifluoroacetic acid was added to remove the Boc group. The crude products were re-precipitated by ethanol and diethyl ether three times to ensure their polydispersity. Gel permeation chromatography (GPC) was used to measure their molecular weights together with the polydispersity index (PDI), and the results are listed in Table 1. As expected, the polymers exhibited degradability induced by the ester bonds, and the molecular weights decreased to ~1 kDa after 1 day in phosphate buffer saline (PBS) buffer solution (Table 1). The degradable capacity may reduce the cytotoxicity and promote the release of DNA cargo during the delivery process. Besides, ^1^H-NMR spectrum of the polymers revealed that the unit ratio of the bridge to PEI were 1.08 and 1.04 respectively, indicating the formation of a linear structure.

### 2.2. The Formation and Properties of Polymer/DNA Polyplexes

**Figure 1:**
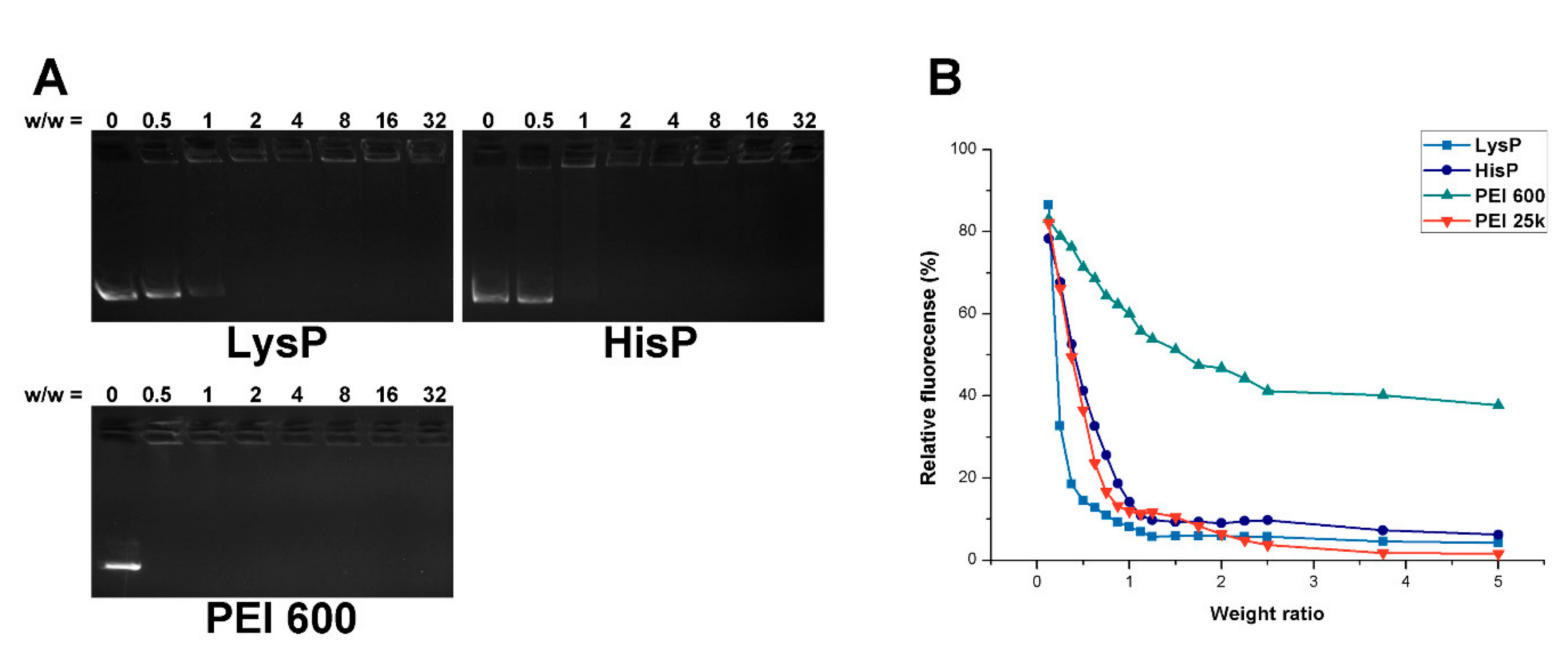
(**A**) Agarose gel electrophoresis of cationic polymers complexed with DNA at different weight ratios. (**B**) Fluorescence quenching assay by the addition of polymers.

The ability of non-viral vectors to bind nucleic acids is the prerequisite to gene delivery. The DNA binding capability of the polymers was investigated by agarose gel electrophoresis. As shown in Figure 1A, these polymers could totally retard DNA migration from the weight ratio (polymer/DNA, *w/w*) of 2, which was slightly higher than PEI 600 Da. We speculate that introduction of the linkers decreased the positive charge density needed for the electrostatic interaction with negatively charged DNA, leading to a slightly higher dosage for full retardation. However, ethidium bromide (EB) exclusion assay revealed that the polymers have stronger DNA binding ability than PEI 600 Da (Figure 1B). The fluorescence of EB intercalating into the base pairs of DNA would be quenched by the addition of other DNA binding agents. The polymers could quench the fluorescence of EB more quickly and thoroughly, indicating their much higher DNA affinity than PEI 600 Da. This could be attributed to their higher molecular weights. Meanwhile, although the polymers have molecular weights less than 10 kDa, they showed a similar fluorescence quenching ability to PEI 25 kDa. Among the polymers, **LysP** showed faster quenching, which might result from the lysine moieties containing strong basic amines.

**Figure 2:**
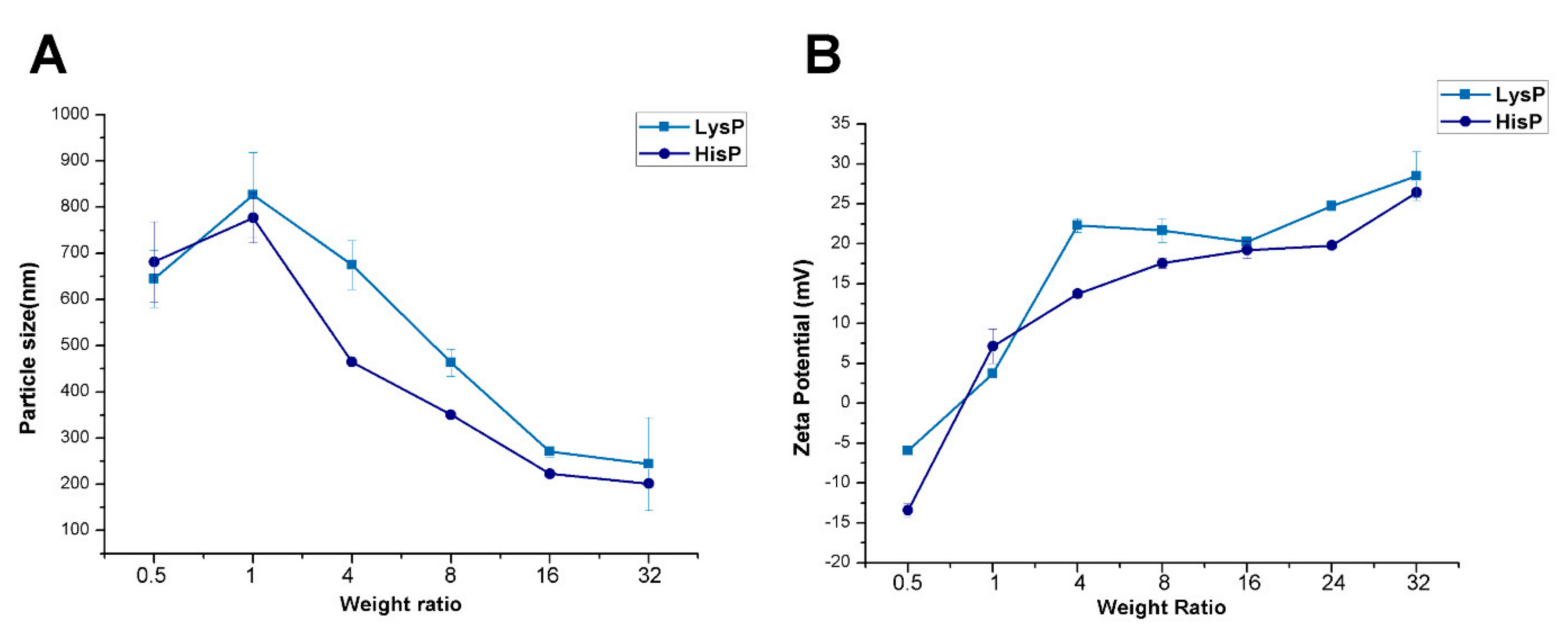
Particle sizes (**A**) and zeta-potentials (**B**) of the polyplexes at various *w/w*.

**Figure 3:**
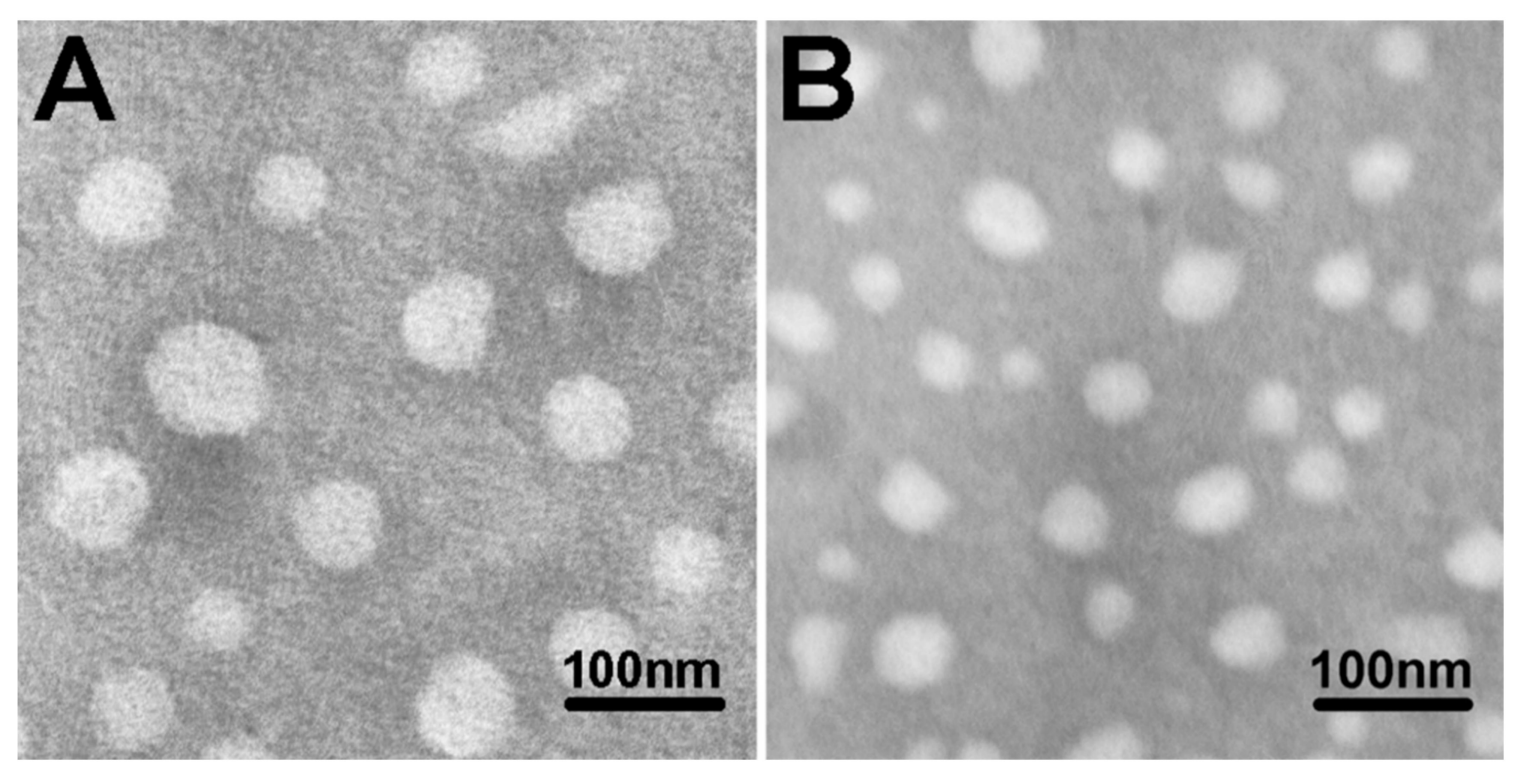
TEM images of **LysP**/DNA (**A**) and **HisP**/DNA (**B**) complexes at their optimal transfection *w/w* (**LysP**: *w/w* = 4; **HisP**: *w/w* = 14) in deionized water.

Appropriate particle size and zeta potential of the polyplexes are also needed for efficient cell internalization [22]. A dynamic light scattering (DLS) experiment was performed to characterize the physical properties of the polyplexes. With the increasing weight ratio of polymer/DNA from 0.5 to 32, the particle size (Figure 2A) and the zeta potential (Figure 2B) ranged from 150~850 nm and −15~+30 mV, respectively, and the trend along with the increase of *w/w* was similar to PEI 25 kDa [13]. At the low weight ratio of 0.5, for the incomplete condensation, the particle size was relatively larger (645 and 681 nm for **LysP** and **HisP,** respectively). The zeta potential of the polyplexes turned positive at the weight ratio of ~1. At this point, the particles were almost neutral, resulting in low electrostatic repulsion and easier aggregation, leading to the largest particle size (826 and 777 nm for **LysP** and **HisP,** respectively). With the further increase of weight ratio, the particle size decreased gradually and tended to be stable at about 200 nm. At the *w/w* of 32, the particle size of **LysP** and **HisP** polyplexes dropped to 170 and 142 nm, respectively. Meanwhile, the zeta potential reached ~+25 mV. Transmission electron microscopy (TEM) was then used to directly visualize the morphology of polyplexes. As shown in Figure 3, both of the two polyplexes could condense DNA into nano-sized spherical particles with the average diameter of 55 ± 15 nm for **LysP** and 37 ± 15 nm for **HisP** under the optimal transfection weight ratio. The smaller particle size measured by TEM compared with DLS might be attributed to the different way to prepare samples. The samples measured by DLS were detected in the hydrated state in solution, while those observed by TEM had been dried after dropped onto carbon-coated copper meshes. The proper physical characteristics of the polyplexes allowed them to be further applied to the gene transfection.

### 2.3. Cytotoxicity

**Figure 4:**
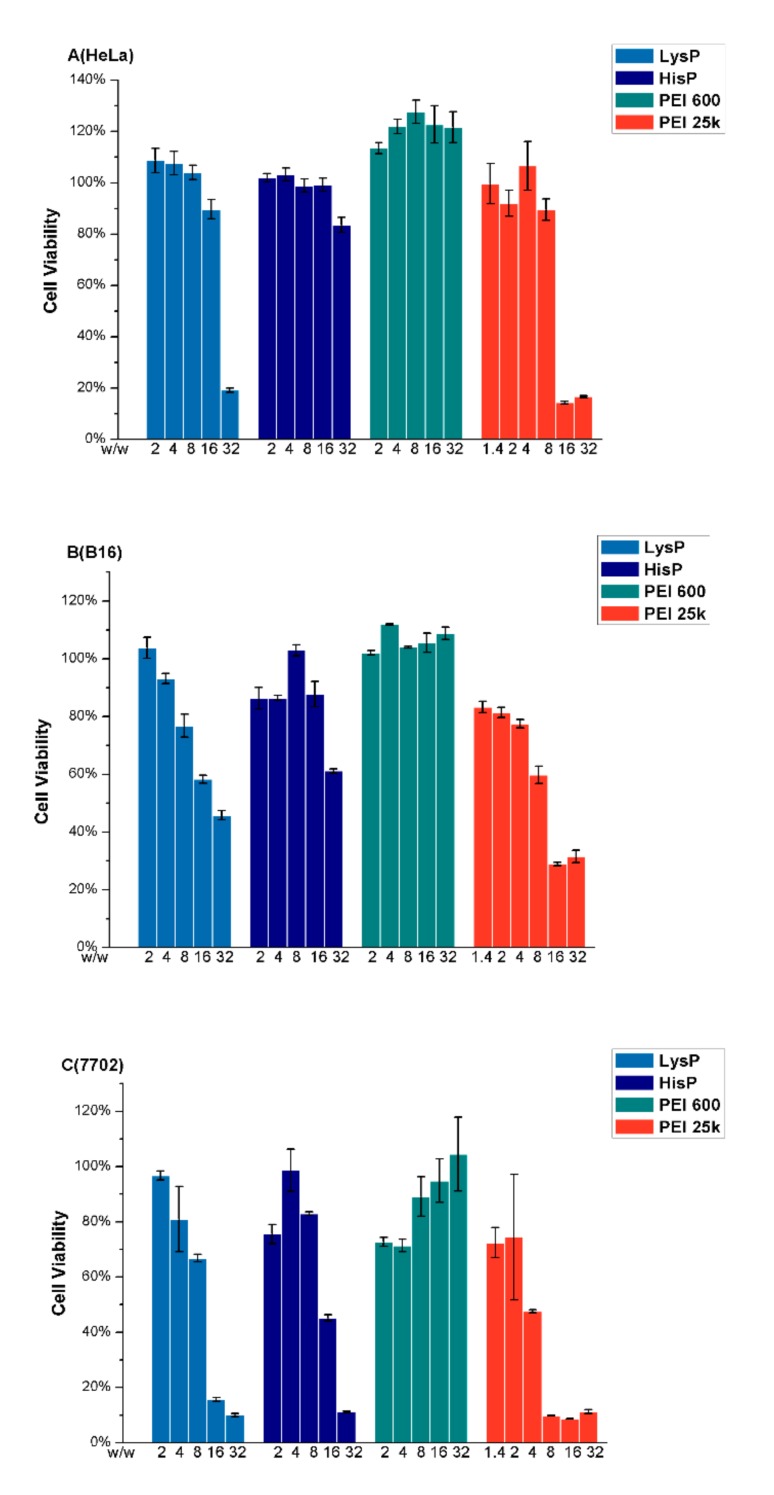
Cytotoxicity of the polyplexes at different weight ratios in HeLa (**A**), B16 (**B**), and 7702 (**C**) cells. PEI 600 Da and PEI 25 kDa (*w/w* = 1.4) were used as control.

To evaluate whether the polymer construction strategy could effectively reduce the cytotoxicity of the polyplexes, MTS assay was applied and the results are shown in Figure 4. The cell viabilities were performed in HeLa, B16 and 7702 cells at various weight ratios, and PEIs with different molecular weights (25 kDa and 600 Da) were used for comparison. Firstly, the viability is different depending on the cell line. In particular, cancer cells grow better than normal cells, and thus can withstand material toxicity. Accordingly, the results show that all materials exhibited higher toxicity in normal cells (7702) than in tumor cells (HeLa and B16). Then, the cell viability decreased with the rise of weight ratio, this could be attributed to the increased positive charge on the polyplex surface. PEI 600 Da showed little toxicity due to its low molecular weight, and on the contrary, PEI 25 kDa showed severe toxicity, especially at higher *w/w*. For the two polymers, **HisP** showed relatively lower toxicity, especially under higher weight ratios. This might be due to its lower molecular weight. For example, in HeLa cells, the cell viability of **LysP** reduced to less than 20% at the *w/w* of 32, but **HisP** could give 84% cell viability at the same weight ratio. In other cell lines, **HisP** also gave higher cell viability than **LysP** at *w/w* of 16 and 32. For PEI 25 kDa, drastic decrease of cell viability was found at *w/w* of 16 for HeLa and B16 cells and eight for 7702 cells, while similar decrease for **LysP** and **HisP** occurred at higher weight ratio, suggesting that the target polymers showed lower toxicity. This might come from their lower molecular weight and degradability, which helps the polymers decompose to smaller molecules.

### 2.4. Gene Transfection Studies

**Figure 5:**
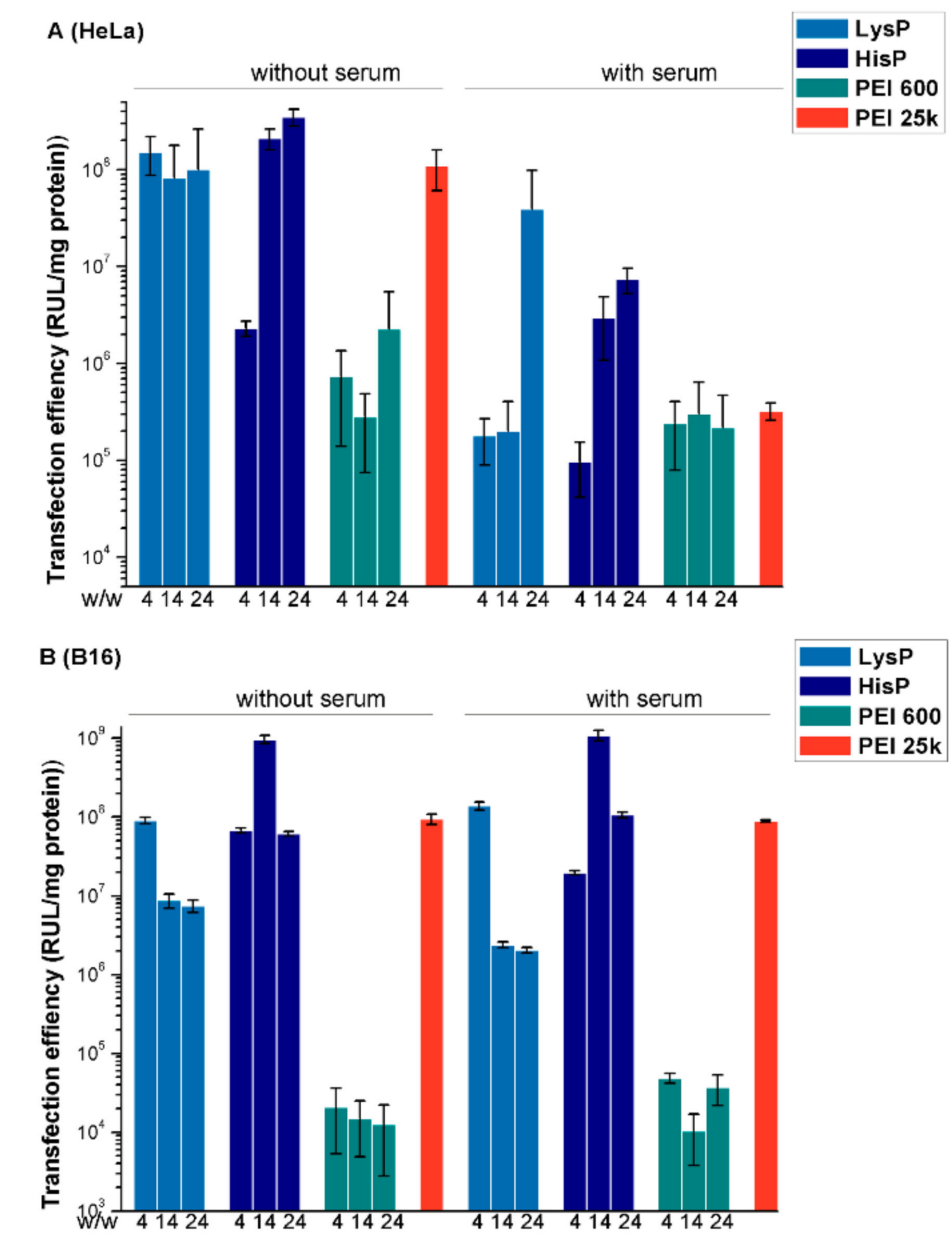

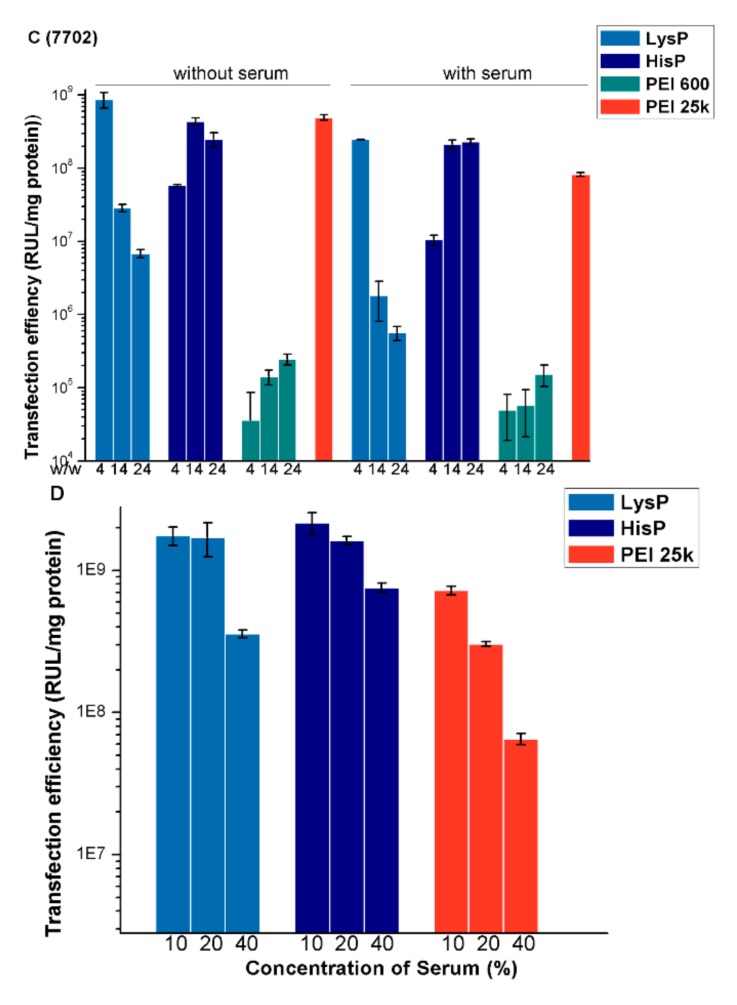
(**A**–**C**) Luciferase gene expression transfected by polyplexes at different weight ratios in different cell lines. (**D**) Effect of serum concentration on the transfection efficiency of **LysP** and **HisP** at their optimal transfection ratios in B16 cells. PEI 25 kDa (*w/w* = 1.4) was used as control.

**Figure 6:**
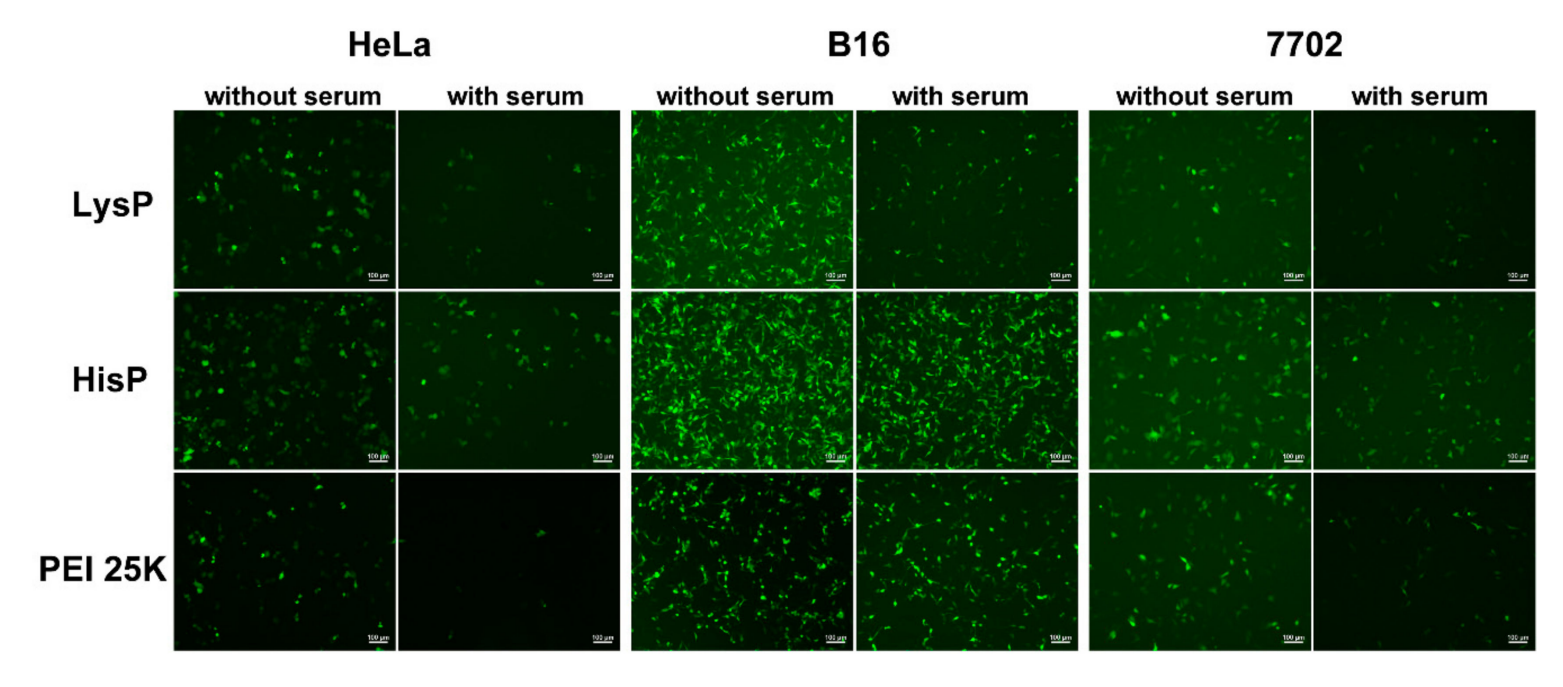
Fluorescence microscope image of pEGFP-transfected cells in the at their optimal weight ratios. PEI 25 kDa (*w/w* = 1.4) was used as control.

The luciferase reporter gene was used to quantitatively assess the transfection efficiency (TE) of the polymers in different cell lines. As shown in Figure 5A–C, although the low molecular weight PEI 600 Da has low toxicity, it could only give poor TE. The transfection weight ratio was screened, and generally, the best TE could be achieved at *w/w* of four and 14 for **LysP** and **HisP**, respectively. The lower optimal weight ratio of **LysP** was probably caused by its larger molecular weight. Besides, its stronger DNA binding ability (Figure 1B) might also impede the DNA release from the polyplex, leading to lower TE at high *w/w*. Although these polymers have lower molecular weights than PEI 25 kDa, they could give higher TE in most cases. In B16 cells, up to 10.2 times higher TE than PEI 25 kDa could be obtained by using **HisP** as vector. Besides, serum seemed to have less negative effect on the TE of the polymers than that of PEI. To confirm this, the luciferase assay was performed with different concentration of serum in B16 cells (Figure 5D). It was found that the TE of PEI 25 kDa reduced rapidly with the increasing concentration of serum, which might be caused by the competitive combination of serum to the positively charged polyplexes. Compared to PEI 25 kDa, the target polymers showed better TE stability, especially for **HisP**, which gave a much less TE decrease with the rise of serum concentration. To directly visualize the gene expression, pEGFP-N1 reporter gene was also used to observe the enhanced green fluorescent protein (eGFP) expressed in different cell lines. The TE could be evaluated by the density of green fluorescence. The images shown in Figure 6 reveal similar results to those in luciferase assay, and more amount of green fluorescent dots could be observed in the transfection by the polyplexes formed from **LysP** and **HisP** whether in the presence of serum or not, proving that these materials could give higher eGFP expression. On the contrary, PEI 600 also mediated poor transfection, and the green fluorescence could be hardly observed (image not shown).

**Figure 7:**
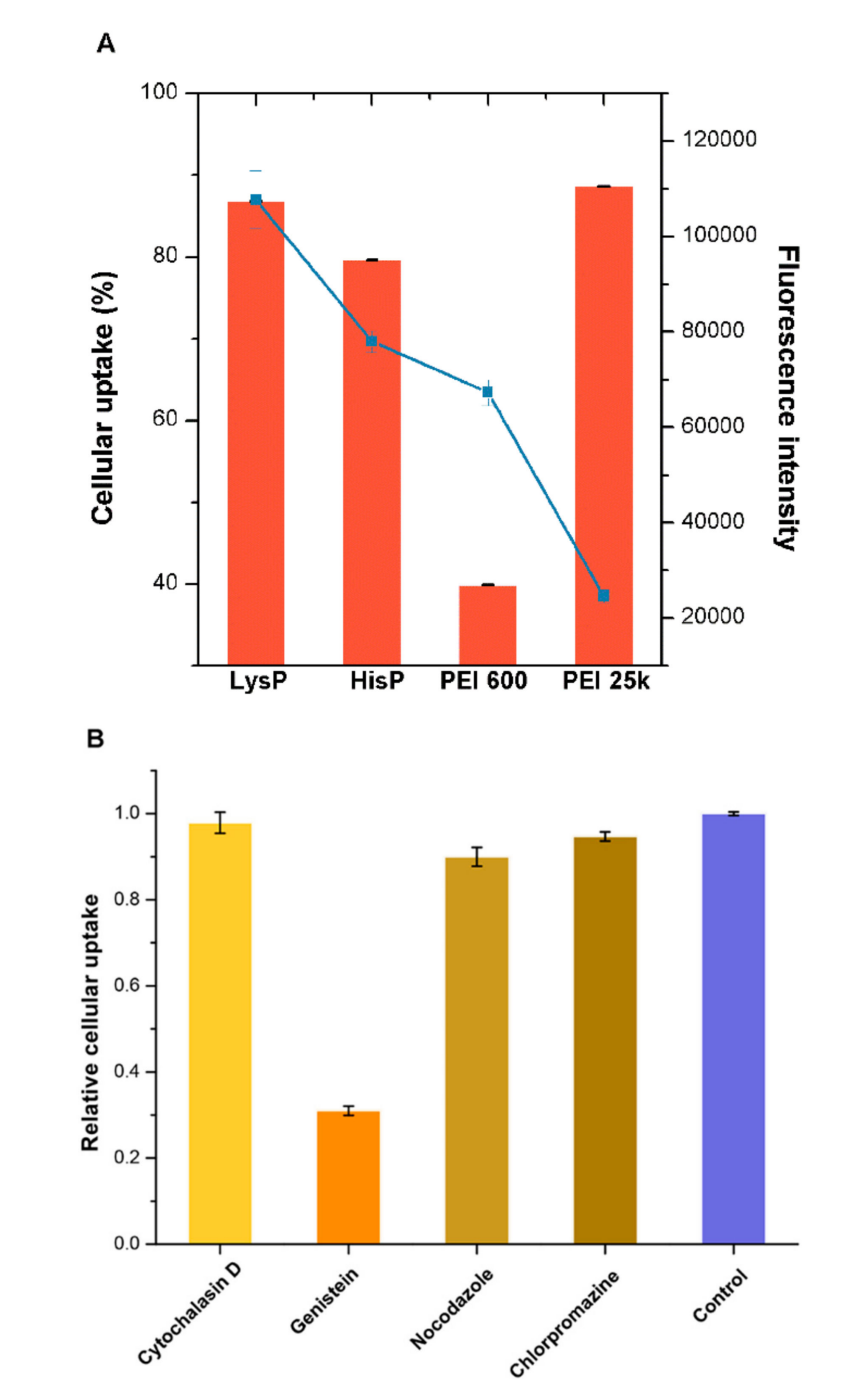
(**A**) Cellular uptake percentage (column) and relative fluorescence intensity (RFI, dot) of polymer/DNA complexes at optimal transfection w/w ratio in B16 cells. PEI 25 kDa (*w/w* = 1.4) was used as control. (**B**) Relative cellular uptake of **HisP**/DNA complexes at the optimal ratio in B16 cells in the presence of various endocytic inhibitors quantified by flow cytometry analysis.

The cellular uptake profiles of these polyplexes were studied by fluorescence activated cell sorting (FACS) analysis in B16 cells. The DNA molecules were labelled with the dye Cy-5 to help to estimate the percentages of fluorescent-positive cells and the relative fluorescence intensity (RFI) after treatment with the polyplexes. As shown in Figure 7A, although the polyplexes formed from **LysP** and **HisP** showed slightly lower uptake cell percentage than those from PEI 25 kDa, the fluorescence intensities of Cy5 induced by them were much higher, suggesting that polymers could medicate endocytosis more effectively, which might be the reason for their higher TE. Further, it is known that the intracellular transport pathway of endocytosed cells is closely related to its endocytic approach, and the endocytic mode depends to a large extent on the delivery system. The complexes without specific target groups mainly electrostatically interact with the negatively charged cells surface, followed by the internalization after being adsorbed on the surface [23]. Hence, the complexes are very likely to enter acidic organelles in cells, and endo/lysosomal degradation is an important barrier for gene transfection [24]. To investigate the endocytosis mechanisms promoted by the polyplexes, four common cellular uptake inhibitors including cytochalasin D, genistein, nocodazole and chlorpromazine were used to figure out the endocytosis pathway of the **HisP**/DNA polyplexes in B16 cells. The results in Figure 7B show that only genistein gave obvious inhibition, and the endocytosis was reduced by 70%. The other three inhibitors showed only slight negative effect. These results indicated that polyplexes entered the cells mainly through caveolae-mediated endocytosis. In this way, the polyplexes could be internalized into cavesomes and then immediately delivered to the Golgi apparatus or endoplasmic reticulum without experiencing endosomal escape, facilitating efficient delivery process [25].

**Figure 8:**
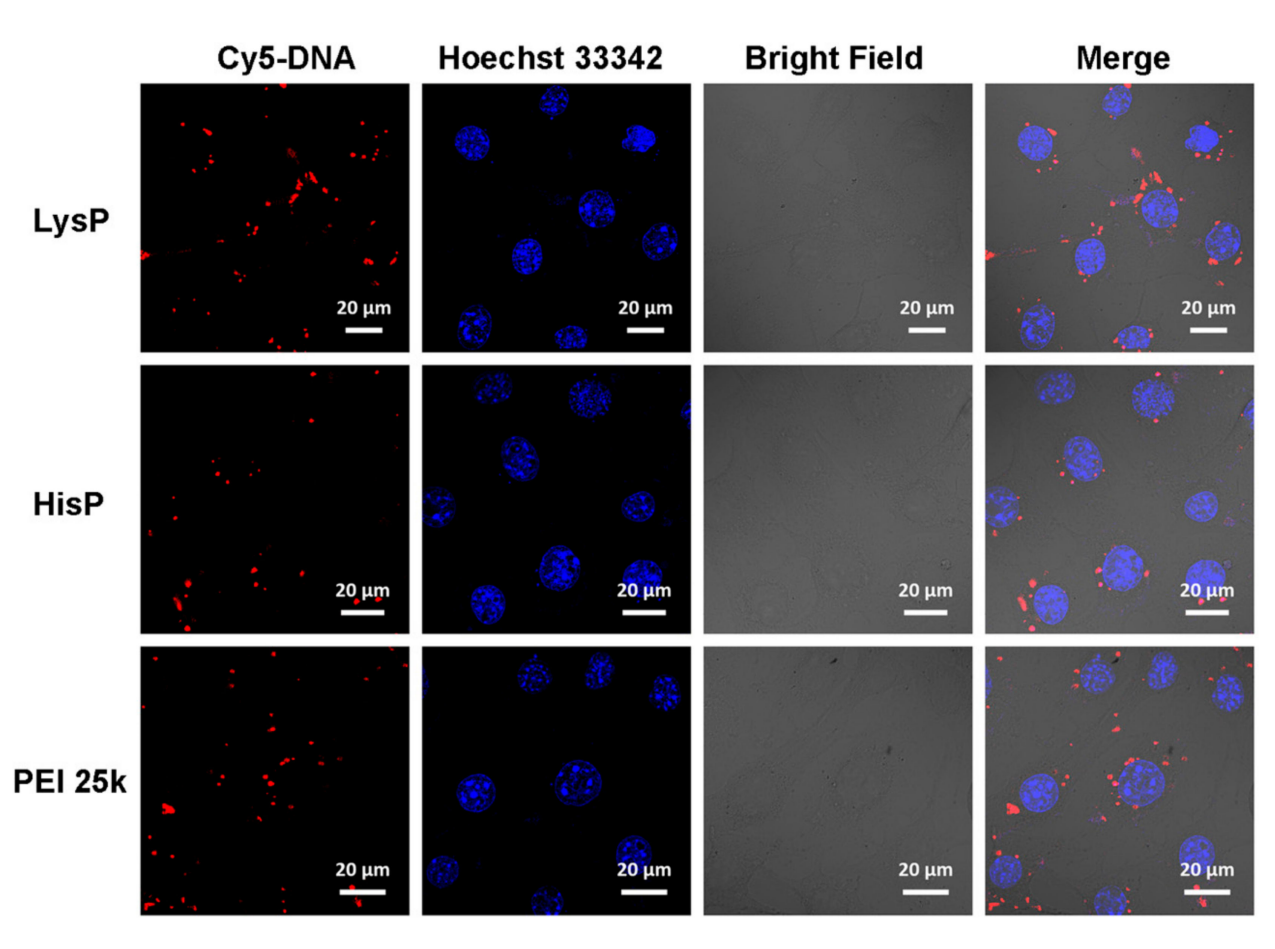
CLSM images of B16 cells transfected with Cy5-labaled DNA by polymers at optimal weight ratio. PEI 25 kDa (*w/w* = 1.4) was used as control.

To visualize the intracellular transport of the complexes in cells, confocal laser scanning microscopy (CLSM) was also carried out to record the distribution of Cy5-labelled DNA (red) in B16 cells. The cell nuclei were stained with hoechst 33342 (blue). As shown in Figure 8, after 4 h incubation, a considerable quantity of Cy5-labelled complexes was localized in the perinuclear region. Compared with PEI 25 kDa, **LysP** gave more Cy5-labled red signal, proving that **LysP**-derived polyplexes owned better endocytosis. These results were consistent with those obtained in the FACS study.

## 3. Experimental Section

### 3.1. Materials and Methods

All chemicals used in experiments were bought from commercial sources and used without additional purification unless otherwise stated. Dichloromethane and methanol were dried with proper desiccants and distilled immediately before use. Column chromatography was performed by 200–300 mesh silica gel or Al_2_O_3_. Deionized water was used to prepare all aqueous solutions. Branched polyethylenimine (bPEI 25 kDa) was purchased from Sigma-Aldrich. The plasmids used in this study re pEGFP-N1 (Clontech, Palo Alto, CA, USA) coding for EGFP DNA and pGL-3 (Promega, Madison, WI) coding for luciferase DNA. The Micro BCA protein assay kit was obtained from Pierce (Rockford, IL, USA). Cy5 was bought from Mirus Bio, LLC (Madison, WI, USA). The luciferase assay kit and MTS (3-(4,5-dimethylthiazol-2-yl)-5-(3-carboxymethoxy-phenyl)-2-(4-sulfophenyl)-2H tetrazolium, inner salt) were purchased from Promega (Madison, WI, USA). Endotoxin-free plasmid purification kit was bought from TIANGEN (Beijing, China). The ^1^H NMR and ^13^C-NMR spectra were measured on a Bruker AM400 NMR spectrometer. Proton Chemical shifts of NMR spectra were given in ppm relative to internals reference TMS (^1^H, 0.00 ppm). HRMS (ESI) spectral data was measured on a Bruker Daltonics Bio TOF mass spectrometer. We used a gel permeation chromatography system (GPC) to measure the molecular weights (*M_w_*) and the polydispersity index (PDI, *M_w_*/*M_n_*) of the polycations. The GPC consisted of a Waters 515 pump, a linear 7.8 × 300 mm column (Waters Corp, Milford, MA, USA), an 18 angle laser scattering instrument (Wyatt Technology Corporation, Santa Barbara, CA, USA), and an OPTILAB DSP interferometric refractometer (Wyatt Technology Corporation, Santa Barbara, CA, USA). 0.5 M NaOAc/0.5 M HOAc (pH 4.6) passed through a 0.02 μm film filter was used as the eluent. The flow rate was 1 mL min^−1^.

### 3.2. Synthesis and Characterization of Compound ***B***

2Boc-Lys was prepared according to the method reported before^21^. 2Boc-Lys (5 g, 14.4 m mol) was dissolved in dichloromethane (DCM), then *N*-methylmorpholine (1.75 g, 17.3 mmol) and isobutyl chloroformate (2.37 g, 17.3 m mol) was add to the solution at 0 °C. After stirring for 0.5 h, diethanolamine (1.82 g, 17.3 m mol) was add to the solution and stirred overnight at room temperature. When the reaction completed, the reaction mixture was washed with saturated aqueous NaHCO_3_ solution, water and brine in sequence. After dried with anhydrous Na_2_SO_4_, the organic layer was distilled under reduced pressure and the residue was purified by silica gel column chromatography (*v/v* 10:1, DCM-CH_3_OH) to give compound **A** as colorless or yellowish thick oil. Yield: 40%. ^1^H-NMR (400 MHz, CDCl_3_, TMS): δ = 1.41, (s, 18H, -Boc), 1.47‒1.88, (m, 4H, -C*H*_2_C*H*_2_CH_2_NHBoc-), 3.06, (m, 2H, -C*H*_2_CH_2_CH_2_CH_2_NHBoc), 3.46, (m, 2H, -C*H*_2_NHBoc), 3.60–3.66, (m, 4H, -CONC*H*_2_CH_2_O-), 3.82, (m, 4H, -CONCH_2_C*H*_2_O-), 4.22, (m, 1H, -C*H*-), 4.59‒4.71, (m, 2H, -O*H*). ^13^C-NMR (CDCl_3_, 100 MHz): δ = 22.3, 28.3, 29.5, 32.3, 39.9, 50.3, 51.9, 60.4, 80.1, 156.1, 174.4. HR-MS (ESI): Calcd for: C_20_H_39_N_3_O_7_: 456.2686 [M + Na]^+^; Found: 456.2686 [M + Na]^+^.

Compound **A** (2 g, 4.61 m mol) and triethylamine (1.12 g, 11.07 m mol) were dissolved in anhydrous DCM. Acryloyl chloride was dissolved in dry DCM and then added dropwise to the stirred solution in the ice bath. The mixture was stirred overnight at room temperature. After that, the mixture was washed by saturated aqueous NaHCO_3_ solution, water and brine in sequence, followed by evaporation of the volatile solvent. The residue was purified with silica gel column chromatography (*v/v* 1:1, PE-EA) to give compound **B** as colorless thick oil. Yield: 59%. ^1^H-NMR (400 MHz, CDCl_3_, TMS): δ = 1.43 (s, 18H, -Boc), 1.25‒1.31 (m, 2H, -NCONHCHCH_2_C*H*_2_-), 1.49‒1.57 (m, 2H, -C*H*_2_CH_2_NH-Boc), 1.62 (m, 2H, -NCONHCHC*H*_2_-), 3.09 (m, 2H, -C*H*_2_NH-Boc), 3.42‒3.92 (m, 4H, -OCH_2_C*H*_2_N-), 4.26‒4.39 (m, 4H, -OC*H*_2_CH_2_N-), 4.66 (m, 1H, -C*H*-), 5.83‒5.88 (m, 2H, -OCC*H*CH_2_), 6.07‒6.18 (m, 2H, -OCCHC*H*_2_), 6.38‒6.44 (m, 2H, -OCCHC*H*_2_). ^13^C-NMR (100 MHz, CDCl_3_): δ = 22.5, 28.4, 33.4, 40.2, 45.6, 47.1, 49.9, 61.8, 79.7, 127.9, 131.5, 156.0, 165.0, 173.1. (HR-MS (ESI): Calcd for: C_20_H_39_N_3_O_7_: 564.2892 [M + Na]^+^; Found: 564.2917 [M + Na]^+^.

### 3.3. Synthesis and Characterization of Compound ***D***

Firstly, 2Boc-His and compound **C** were prepared according to the method reported before [21,26]. Then, the protecting t-butyloxycarbonyl (Boc) group of compound **C** (5 g, 16.12 m mol) was removed by trifluoroacetic acid (TFA) at 0 °C in the DCM solution. After removing the solvent and most TFA, the residues were dissolved in 50 mL of DCM. In total, 5 mL of NH_3_·H_2_O was dissolved in 50 mL of water and added to the DCM solution until the pH of organic phase reached ˃7. The organic phase was separated from mixture and dried with anhydrous Na_2_SO_4_. Then, 2Boc-His (4.60 g, 13.29 m mol), 1-hydroxybenzotriazole (2.03 g, 13.29 m mol) and *N*, *N*-diisopropylethylamine (1.72 g, 13.29 m mol) were added to the organic phase. 1-Ethyl-3-(3-dimethyl propyl) carbodiimide was dissolved in dry DCM and then added dropwise to the stirred solution at 0 °C. After stirring overnight, the mixture was washed by saturated NaHCO_3_ solution, water and brine in sequence, followed by evaporation of the volatile solvent. The residue was purified with silica gel column chromatography (*v/v* 1:1, PE-EA) to give compound **D** as white or yellowish powder. Yield: 13.0%. ^1^H-NMR (400 MHz, CDCl_3_, TMS): δ = 1,39 (s, 9H, -CHNHBoc), 1.59 (s, 9H, -NBoc), 2.83–2.91 (m, 4H, -NH_2_CHC*H*_2_-), 3.45‒3.85 (m, 4H, -OCH_2_C*H*_2_N-), 4.28 (m, 4H, -OC*H*_2_CH_2_N-), 4.96‒4.98 (m, 1H, -C*H*NH-), 5.79‒5.86 (m, 2H,-OCC*H*CH_2_), 6.06‒6.16 (m, 2H, -OCCHC*H*_2_), 6.37‒6.41 (m, 2H, -OCCHC*H*_2_), 7.15 (s, 1H, -C*H*NBoc), 7.96 (s, 1H, -NC*H*NBoc). ^13^C-NMR (100 MHz, CDCl_3_): δ = 27.9, 32.5, 47.3, 49.8, 62.1, 79.7, 85.4, 114.7, 128.0, 131.4, 136.8, 138.6, 146.9, 155.0, 165.8, 172.4. HR-MS (ESI): Calcd for: C_20_H_39_N_3_O_7_: 573.25331 [M + H]^+^; Found: 573.2566 [M + H]^+^.

### 3.4. Synthesis and Characterization of Target Polymers ***LysP*** and ***HisP***

Briefly, bPEI 600 (300 mg, 0.5 m mol) and compound **B** or **D** (0.5 m mol) were separately dissolved in 1 mL of anhydrous methanol and 1 mL of anhydrous DCM. The reaction mixtures were refluxed at 45 °C with constant stirring for 72 h. After that, the mixtures were diluted with 20 mL of DCM, and 5 mL of TFA was added with stirring overnight to remove the protecting Boc group of polymers. After removing the solvent and most TFA, the residues were dissolved in 1 mL of ethanol and precipitated by the addition of diethyl ether. The precipitation was collected and dried in vacuum to get the product as yellow viscous solid. The molecular weights of **LysP** and **HisP** were measured by GPC.

**LysP**. 58.5% yield. Mw: 9115 Da, PDI: 1.97. ^1^H-NMR (400 MHz, D_2_O, TMS): δ = 1.27‒1.29, (m, 2H, -NCONHCHCH_2_C*H*_2_-), 1.50‒1.52, (m, 2H, -CCH_2_CH_2_C*H*_2_CH_2_NH_2_), 1.72‒1.74, (s, 2H, -NCONHCHC*H*_2_-), 2.55‒3.36, (m, 54H, -NHC*H*_2_CH_2_N- and - CCH_2_CH_2_CH_2_C*H*_2_NH_2_),

**HisP**. 52.7% yield. Mw: 7208 Da, PDI: 1.79. ^1^H-NMR (400 MHz, DMSO, TMS): δ = 2.48–3.47 (m, 60H, -NHC*H*_2_CH_2_N- and -NH_2_CHC*H*_2_C-), 7.40 (s, 1H, -NHC*H*C-), 9.0, (m, 1H, -NHC*H*N-).

### 3.5. Agarose Gel Retardation Assay

**LysP**/DNA and **HisP**/DNA complexes at different weight ratios ranging from 0.5 to 3.2 were prepared by adding an appropriate volume of **LysP**/**HisP** to 5 μL of pUC-19 (0.025 mg/mL). The obtained complex solutions were diluted to 10 μL, and then all the complexes were incubated at 37 °C for 30 min. Then, the complexes were electrophoresed on a 1% (*w/v*) agarose gel containing GelRed™ and with Triseacetate (TAE) running buffer at 140 V for 30 min. DNA was visualized under an ultraviolet lamp using a Vilber Lourmat imaging system.

### 3.6. Ethidium Bromide Displacement Assay

The ability of **LysP** and **HisP** to condense DNA was studied by using EB exclusion assays. Fluorescence spectra were measured at room temperature in air by a Horiba Jobin Yvon Fluoromax-4 spectrofluorometer and corrected for the system response. EB (2.5 mL, 1 mg/mL) was added into quartz cuvette containing 2.5 mL of 10 mM Hepes solution. The fluorescence intensity of EB was measured after enough shaking. Then CT DNA (10 mL, 1 mg/mL) was added to the solution and mixed symmetrically, and the measured fluorescence intensity was the result of the interaction between DNA and EB. Subsequently, the solution of polymers (1 mg/mL, 2 mL for each addition) was added to the above solution for further measurement. All the samples were excited at 520 nm and the emission was measured at 600 nm. The pure EB solution and DNA/EB solution without cationic polymer were respectively used as negative and positive controls. The percent relative fluorescence (%F) was determined using the equation %F = (F−F_EB_) / (F_0_−F_EB_), wherein F_EB_ and F_0_ denote the fluorescence intensities of pure EB solution and DNA/EB solution, respectively.

### 3.7. Particle Size and ζ-Potential Measurement in Water

Zeta potential (ζ-potential) and size of the polyplex particles were measured by Nano-ZS ZEN3600 apparatus (Malvern Instruments) at 25 °C. The complexes with various weight ratio of polycations were prepared by adding 1 mg of pUC-19 to appropriate volume of the deionized water. Before measurement, the solution of the complexes was incubated at 37 °C for 0.5 h and then diluted with deionized water to 1 mL. The data were shown as mean ± standard deviation (SD) based on triplicate independent measurement.

### 3.8. Transmission Electron Microscopy (TEM)

The morphologies of the polyplexes were observed by TEM (JEM-100CXa) with an acceleration voltage of 100 kV. 1 mg of pUC-19 was added to the appropriate volume of the polymer solution (optimal weight ratio of each sample), then diluted to the total volume of 50 μL. The solution of the polyplexes was incubated at 37 °C for 0.5 h. The polyplex solution was diluted with deionized water to 1 mL before measurement. A drop of DNA/polymer complexes solution was placed onto the copper grid. The excess solution was blotted away with filter paper after a few minutes. Then, a drop of 0.5% (*w/v*) phosphotungstic acid was placed on the above grid. The grid was dried at room temperature for several minutes before observation.

### 3.9. Cell Culture

HeLa cells were cultured in Dulbecco’s modified Eagle’s medium (DMEM) with 10% fetal bovine serum (FBS) and 1% antibiotics (penicillin-streptomycin, 10 KU mL^−1^) at 37 °C in a humidied atmosphere containing 5% CO_2_. B16 and 7702 cells were cultured in Roswell Park Memorial Institute-1640 (RPMI-1640) with 10% fetal bovine serum (FBS) and 1% antibiotics (penicillin-streptomycin, 10 KU mL^−1^) at 37 °C in a humidied atmosphere containing 5% CO_2_.

### 3.10. Amplification and Purification of Plasmid DNA

pGL-3 plasmids were transformed in M109 Escherichia coli and seed as the luciferase reporter gene. pEGFP-N1 plasmids is the enhanced green fluorescent protein reporter gene which was transformed in E. coli DH5a. Both plasmids were amplified in E. coli grown in LB medium at 37 °C and 220 rpm overnight. The plasmids were purified by an EndoFree TiangenTM Plasmid Kit. Then, the purified plasmids were dissolved in TE (Tris + EDTA) buffer solution and stored at −80 °C. The integrity of plasmids was confirmed by agarose gel electrophoresis. The purity and concentration of plasmids were determined by the ratio of ultraviolet (UV) absorbances at 260–280 nm.

### 3.11. Gene Transfection Assay

Gene transfection of a series of complexes was investigated in HeLa, B16 and 7702 cells. Cells were seeded in 48-well plates (5 × 10^4^ cells/well) and grown to 70%–80 % cell confluence at 37 °C for 24 h in 5 % CO_2_. Before transfection, the medium was exchange into a serum-free or a serum-containing culture medium containing polymer/pDNA (0.4 μg) complex at various weight ratios. The medium was replaced with fresh medium containing serum and incubated for another 20 h after 4 h standard incubator conditions.

For fluorescent microscopy assays, cells were transfected by complexes containing pEGFP-N1. After 24 h incubation, the cells expressed pEGFP-N1 were observed with an inverted fluorescence microscope (Nikon Eclipse TE 2000E, Tokyo, Japan) equipped with a cold Nikon camera. Control transfection was performed in each case using a commercially available transfection reagent bPEI 25 kDa based on the standard conditions specified by the manufacture.

For luciferase assays, cells were transfected with complexes containing pGL-3 plasmids. After 24 h transfection as described before, the luciferase assay was performed according to the manufacturer’s protocols (Promega). The luciferase activity was measured by microplate reader (Model 550, BioRad, Hercules, CA, USA). The protein content of the lysed cell was determined by BCA protein assay kit (Pierce). Gene transfection efficiency was expressed as the relative fluorescence intensity per mg protein (RLU/mg protein). bPEI 25 kDa and bPEI 600 Da were used as control. All experiments were performed in triplicate.

### 3.12. Cell Viability Assay

Toxicity of **LysP** and **HisP** toward HeLa cells, B16 cells and 7702 cells was determined by an MTS reduction assay. The cells mentioned above were seeded 1 × 10^4^ cells/well into 96-well plates for 24 h at 37 °C in a humidied atmosphere containing 5% CO_2_. The medium was replaced with 100 mL of fresh medium without FBS, to which 100 mL complexes at various weight ratio of polymer relative to pDNA was added to achieve final volume of 200 mL. The cells were then incubated in the medium without FBS containing polymer/pDNA (0.2 mg) complexes at various weight ratios. The polyplexes solutions were removed after 24 h of incubation. 20 mL of MTS and 80 mL of PBS were added to each well for extra 1 h incubation. In the measurement of B16 cells, RPMI-1640 medium was used instead of PBS. The absorbance was measured by a microtiter plate reader. The cell viability (%) was obtained according to the manufacturer’s instructions as follows: cell viability = (ODtreated/ODcontrol) × 100%. The bPEI 25 kDa and bPEI 600 Da were used as control. All experiments were performed in triplicate.

### 3.13. Cellular Uptake of Plasmid DNA

The cellular uptake of the polymer/Cy5-labeled DNA complexes was analyzed by flow cytometry. The Label IT Cy5 Labeling Kit was used to label pDNA with Cy5 according to the manufacturer’s protocol. The cells mentioned were cultured 1 × 10^5^ cells/well in 24-well plates for 24 h at 37 °C in a humidied atmosphere containing 5% CO_2_ before in vitro gene transfection. Then, the B16 cells were incubated with the Cy5-labeled polyplexes (0.8 μg DNA/well, optimal weight ratio of each sample) for 4 h at 37 °C in different serum-containing medium and uptake efficacy was analyzed using flow cytometry. After that, the cells were washed with 1 × PBS and harvested with 0.25% Trypsin/EDTA and resuspended in 1 × RPMI-1640. The uptake of Cy5-labeled plasmid DNA was measured in the FL4 channel using the red diode laser (633 nm). Mean fluorescence intensity was analyzed using flow cytometer (Becton Dickinson and Company, Franklin Lakes, NJ, USA).

### 3.14. Confocal Laser Scanning Microscopy (CLSM) Analysis

To investigate the cellular uptake of the complexes, Cy5-labeled DNA was used to monitor intracelluar trafficking behaviors (0.8 μg DNA/well, optimal weight ratio of each sample). The B16 cells were incubated with the polyplexes for 4h. The nuclei of B16 cells were stained with hoechst 33342. Co-localizations of the polyplexes with acidic vesicles in B16 cells were observed by the CLSM observation which was performed using LSM 780 (Zeiss) at excitation wavelengths of 405 nm for hoechst 33342 (blue), 633 nm for Cy5 (red).

### 3.15. Biodegradation of Polymers

**LysP** and **HisP** was dissolved in 1 × PBS buffering solution and incubated in the shaker incubator at 37 °C, 100 rpm. The polymer was sampled at different time points and lyophilized. The *M_w_* and PDI was measured by GPC.

## 4. Conclusions

In summary, two novel polycations were constructed via Michael addition from LMW PEI 600 Da and amino acid-containing linkages. These polymers could efficiently condense DNA into nanoparticles with appropriate particle size and zeta potential. Several assays were performed to confirm their degradation ability, good gene transfection efficiency and low cytotoxicity. Compared with the “golden standard” PEI 25 kDa, up to 10.2 times higher transfection efficiency could be obtained. Besides, good serum tolerance was also achieved for these vectors. Mechanism study revealed that the polyplexes enter the cells mainly through caveolae-mediated endocytosis pathway. Together with their biodegradable ability, such a pathway facilitates the internalization of polyplexes and the release of DNA. These polycations are expected to be promising candidates for non-viral gene delivery.

## Figures and Tables

**Table 1:** Molecular weight of polymers and their degradability.

Polymers	*M_w_* (Da)	PDI	*M_w_* After 24 h of Degradation (Da)	*M_w_* After 48 h of Degradation (Da)
**LysP**	9115	1.97	1091	1095
**HisP**	7208	1.79	1085	1093

## Sample Availability

Samples of the compounds are not available from the authors.
